# Correlation analyses between MIBG myocardial scintigraphy and monoamine levels in dementia with Lewy bodies show potential link with the serotonergic system

**DOI:** 10.1016/j.prdoa.2025.100346

**Published:** 2025-05-12

**Authors:** Annelies Heylen, Yannick Vermeiren, Sebastiaan Engelborghs, Frank Van Acker, Peter Paul De Deyn, Debby Van Dam

**Affiliations:** a Laboratory of Neurochemistry and Behaviour, Experimental Neurobiology Unit, Department of Biomedical Sciences, University of Antwerp, Antwerp, Belgium; b Division of Human Nutrition and Health, Chair Group of Nutritional Biology, Wageningen University & Research (WUR), Wageningen, the Netherlands; c Faculty of Medicine & Health Sciences, Translational Neurosciences, University of Antwerp, Antwerp, Belgium; d Department of Neurology, Universitair Ziekenhuis Brussel (UZ Brussel), Brussels, Belgium; e Neuroprotection and Neuromodulation (NEUR) Research Group, Center for Neurosciences, Vrije Universiteit Brussel, Brussels, Belgium; f Department of Nuclear Medicine, Ziekenhuis Aan de Stroom (ZAS) Middelheim, Antwerp, Belgium; g Department of Neurology and Alzheimer Research Center, University of Groningen and University Medical Center Groningen, Groningen, the Netherlands

**Keywords:** DLB, UPLC, Biogenic amines, Serotonin, Dopamine, Noradrenaline, Norepinephrine, H/M ratio, 5-HIAA, 5-HT

## Abstract

**Setting:**

Dementia with Lewy bodies (DLB) remains poorly understood and frequently misdiagnosed, complicated by co-pathology with other dementia forms. DLB patients often present with autonomic dysfunction and peripheral Lewy body pathology alongside central lesions. Monoaminergic neurotransmitter systems seem an early target for DLB pathology, especially the noradrenergic system. Noradrenaline analogue ^123^I-metaiodobenzylguanidine (MIBG) is considered an indicative biomarker for peripheral noradrenergic sympathetic denervation.

**Objectives:**

Our aim was to measure paired monoaminergic levels and MIBG scintigraphy values in DLB patients, exploring a possible link between noradrenergic neurotransmission and peripheral denervation.

**Design:**

44 patients with a possible DLB diagnosis entered the study. Peripheral uptake of ^123^I-MIBG was determined by the heart-to-mediastinum (H/M) ratio, as a measure for noradrenergic sympathetic denervation. In cerebrospinal fluid (CSF), serum and plasma samples, monoamines ((nor)adrenaline ((N)A), 5-hydroxytryptamin (5-HT, serotonin), dopamine (DA)) and respective metabolites (3-methoxy-4-hydroxyphenylglycol (MHPG), 5-hydroxyindoleacetic acid (5-HIAA), homovanillic acid (HVA) and 3,4-dihydroxyphenylacetic acid (DOPAC)), were measured by means of reversed-phase ultrahigh-performance liquid chromatography with electrochemical detection.

**Results:**

We found significant correlations between the H/M ratio and serum 5-HIAA, plasma 5-HT, plasma 5-HIAA/5-HT and plasma HVA/5-HIAA, but no further correlations with the noradrenergic system. CSF-serum MHPG, CSF-serum DOPAC, CSF-serum HVA, CSF-plasma MHPG, CSF plasma NA, CSF-plasma DOPAC, CSF-plasma MHPG/NA and CSF-plasma DOPAC/DA were significantly correlated.

**Conclusions:**

These results show an association between the H/M ratio and serotonergic system, but not between peripheral noradrenergic denervation and circulating noradrenergic levels.

## 1 Introduction

Dementia with Lewy bodies (DLB) is one of the most common forms of dementia after Alzheimer’s disease (AD) [1]. DLB is pathologically characterized by the intraneuronal aggregation of alpha-synuclein, and, to a lesser extent, ubiquitin and hyperphosphorylated tau proteins, into inclusions called Lewy bodies [2]. Pathological accumulation of these Lewy bodies results in a wide range of cognitive and behavioral symptoms associated with dementia. DLB patients also often present with symptoms related to autonomic dysfunction and display peripheral Lewy body pathology alongside central lesions [3].

^123^I-metaiodobenzylguanidine (MIBG) myocardial scintigraphy is used in DLB as an indicative biomarker for peripheral noradrenergic sympathetic denervation [4]. With this imaging technique, the uptake of the radioactive tracer and noradrenaline (NA) analogue ^123^I-MIBG between heart and mediastinum is determined. The heart-to-mediastinum (H/M) ratio then serves as a measure for noradrenergic sympathetic innervation [5]. Reduced uptake ^123^I-MIBG is thought to reflect peripheral noradrenergic denervation, which could be associated with the autonomic dysfunction often experienced by DLB patients. Due to the overlap in clinical symptoms, DLB patients often receive a differential diagnosis of AD [6]. However, previous research indicates that ^123^I-MIBG myocardial scintigraphy might have an added value for the differential diagnosis of DLB versus AD [[7], [8], [9]].

Current research also points to a role for monoamine neurotransmitter systems in the disease progression of DLB. Monoamine neurotransmitters, such as NA, adrenaline (A), dopamine (DA) and 5-hydroxytryptamin or serotonin (5-HT), are involved in many neurophysiological processes, including behavior, cognition and autonomic function [[10], [11], [12]]. Interestingly, NA, DA and 5-HT cell bodies originate from the brainstem, an early target for both Lewy body and tau pathology [2,13,14]. As a consequence, monoaminergic neurotransmission is significantly altered in DLB [[15], [16], [17]]. Correspondingly, monoaminergic alterations have also been linked to cognitive decline, behavioral disturbances and autonomic dysfunction in neurodegenerative disorders [[17], [18], [19],^46^]. More specifically, the locus coeruleus, the central noradrenergic nucleus and production site for NA in the brain [20], appears to be one of the earliest, most vulnerable targets of Lewy body pathology [19,21]. This results in altered noradrenergic activity in early stages of DLB, which could aid in the discrimination of DLB from other types of dementia, such as AD or Parkinson’s disease (dementia) (PD(D)). For example, we previously found that the levels of NA metabolite 3-methoxy-4-hydroxyphenylglycol (MHPG) were significantly different in brains of DLB and AD patients [18]. A later study by Van der Zee et al. [16] found that cerebrospinal fluid (CSF) MHPG was significantly higher in DLB compared to cognitively normal PD or control individuals, as were the levels of 5-hydroxyindoleacetic acid (5-HIAA), the main 5-HT metabolite. In addition, their results showed that both CSF/serum MHPG levels and the serum MHPG/NA metabolic turnover ratio could accurately discriminate DLB from PDD patients. Janssens et al. [15] demonstrated significant differences in CSF, serum MHPG and MHPG/NA ratio between DLB and AD patients. In conclusion, these findings underline the need to further investigate monoaminergic dysfunction in DLB.

Considering a possible link between peripheral noradrenergic denervation and altered noradrenergic neurotransmission in DLB, our primary goal was to measure paired noradrenergic levels in blood and CSF and MIBG scintigraphy values in these patients. Dopaminergic and serotonergic compounds were also investigated, given the selective neurodegeneration of these other brainstem monoaminergic nuclei in DLB. The inclusion of both CSF, serum and plasma samples allows for a comprehensive investigation of the correspondence in monoaminergic levels between CSF and blood, as well as between serum and plasma. Monoaminergic compounds may behave differently in serum or plasma, e.g. due to enzymatic activity, degradation or binding to platelets, proteins or clotting factors. Including both samples types will increase our understanding of the behavior of these potential biomarkers and allow us choose the most reliable and reproducible matrix. Ultimately, a strong link between specific monoaminergic compounds and DLB-related pathology could signify an added value for monoaminergic neurotransmitters as discriminative biomarkers. The use of serum and plasma measures whether or not combined with MIBG could provide a less invasive, cost- and time-effective alternative for CSF collection through lumbar puncture.

## 2 Methods

### 2.1 Study population

Samples were obtained from the NeuroBiobank of the Institute Born-Bunge (Wilrijk, Antwerp, Belgium; ID: BB190113). Patients were recruited at the Memory Clinic of the Hospital Network Antwerp (ZNA) Middelheim and Hoge Beuken as part of their diagnostic clinical workup. The study population consists of patients who received a clinical diagnosis of possible DLB at the Memory Clinic between 2006 and 2013 (n = 85), and in whom ^123^I-MIBG myocardial scintigraphy was performed to explore its discriminative potential for a DLB versus AD differential diagnosis [7]. For this study, the cohort was reduced to a total of 44 patients for which linked CSF and blood samples were also available. The clinical diagnosis was based on the diagnostic criteria described by McKeith et al. [22]. In addition, patients were stratified by ^123^I-MIBG status. Patients with a H/M value lower than 1.68 were considered MIBG-positive, based on the cut-off values as defined by Yoshita et al. [5]. Applying the more recent diagnostic criteria, possible DLB patients with a positive MIBG status may receive a probable DLB diagnosis [23]. No control group is included. This study was approved by the Medical Ethical Committee of the Middelheim General Hospital (Antwerp, Belgium; approval numbers 2805 and 2806) and conducted in compliance with the Helsinki Declaration.

### 2.2 Sample extraction and preparation

Prior to CSF sampling, patients fasted overnight and applied abstinence from smoking for at least 12 h. The CSF samples were obtained by applying a lumbar puncture (LP) in supine position at the intervertebral space L3/L4 or L4/L5 between 8:00 and 10:00 AM. The total amount of CSF (16 mL) was collected in 5 fractions using polypropylene vials (Nalgene; VWR, Leuven, Belgium) and was done in following order: C1 (4.5 mL), C2 (1.5 mL), C3 (1.5 mL), C4 (4.5 mL) and C5 (4.5 mL). Liquid nitrogen was used to freeze fractions C1-C4 on-the-spot, while fraction C5 was centrifuged for 10 min at 3,000 RPM (Centrifuge 5702, rotor A-4-38; Eppendorf, Hamburg, Germany). The supernatant was distributed in polypropylene vials and also frozen in liquid nitrogen. For the experiment, the C5 fraction was used. Blood sampling was performed by venous puncture and the total amount of blood was collected in two serum gel tubes with clotting factor (S-Monovette® 7.5 mL Z-gel subtype; Sarstedt, Numbrecht, Germany) and the samples were centrifuged for 10 min at a rotation speed of 3,000 RPM. The serum was subsequently distributed into marked polypropylene vials and frozen in liquid nitrogen. Plasma samples were drawn on the same day and collected in EDTA tubes. The NeuroBiobank of the Institute Born-Bunge (Antwerp, Belgium) ensured storage of all the samples at a temperature of −80 °C until the start of the analysis.

### 2.3 ^123^I-metaiodobenzylguanidine myocardial scintigraphy

During this procedure, a body scan was performed by using MIBG-scintigraphy as described by Slaets et al. [7]. This was conducted at the Department of Nuclear Medicine of the Hospital Network Antwerp (ZNA) (Middelheim, Antwerp, Belgium). A total of 500 mg of potassium perchlorate was administered to induce a thyroid blockade. This was followed by an intravenous injection of 2 mCi of MIBG. In addition, planar thorax images were taken 4 h after the administration of the ^123^I-MIBG tracer. The data was acquired by using a Philips XCT-scanner and a Varicam (GE) scanner. Both cameras had a high-resolution collimator for low energies, very similar hardware characteristics (LEHR collimator and a large field double-head camera) and the same settings for the acquisition parameters (energy spectrum, pixel size and scan duration). The data was stored in a 128 × 128 matrix size. Myocardial uptake of MIBG tracer was determined by calculating the delayed H/M uptake ratio. This was done by defining two regions of interest over the heart and mediastinum, whereby the ratio is calculated by the ratio of count per pixel in each of the two regions of interest. In this way potential variations, caused by the use of two distinct cameras, were reduced.

### 2.4 Reversed-phase ultra-high-performance liquid chromatography

Neurochemical analysis of plasma monoamine neurotransmitters (NA, A, DA, 5-HT) and their corresponding metabolites (MHPG, 3,4-dihydroxyphenylacetic acid (DOPAC), homovanillic acid (HVA) and 5-HIAA), was performed via an Alexys Neurotransmitter Analysis device (Antec Leyden BV, Zoeterwoude, the Netherlands). This setup consists of a reversed-phase ultra-high-performance liquid chromatography (RP-UHPLC) system, coupled to a Decade II electrochemical detection (ECD) device and equipped with a SenCell with glassy carbon working electrode (2 mm) and in-situ Ag/AgCl reference electrode (ISAAC) at a potential setting of 670 mV and 5nA output range (Antec Leyden BV). The LC110S pump delivered backpressures of an approximate 455–460 bar and maintained an isocratic flow of 62 µL/min. Monoamines and metabolites were separated by means of a short 15 cm Waters Acquity column (BEH C18, 1 mm diameter, particle size 1.7 µm; Waters, Etten-Leur, the Netherlands). Column temperature in the Faraday oven was kept at 37 °C. With a mobile phase consisting of 120 mM citric acid, 120 µM phosphoric acid, 600 mg/L octane-1-sulfonic acid sodium salt (OSA), 8 mM KCl, 0.1 µM ethylenediaminetetraacetic acid (EDTA), 11 % methanol at pH 3.0, optimal compound separation was achieved. Output data were analyzed via Clarity software (DataApex Ltd., v8.1, 2018, Prague, The Czech Republic).

The first step of sample preparation consisted of two consecutive washing cycles during which Amicon Ultra Centrifugal filters (3 kDA; Millipore, Ireland) were washed by centrifugation (14,000 x g (4 °C), 25 min) with 450 μL of sample preparation buffer (without methanol). Then, either 250 µL of CSF or 450 µL of serum or plasma was transferred to the washed Amicon filters and centrifuged (40 min, 14,000 x g, 4 °C). From the resulting filtrate, a 1:7 diluted and undiluted CSF, serum or plasma fraction was automatically injected onto the BEH C18 column via an Alexys AS110 Autosampler (5 μL sample loop) (Antec Leyden BV). Total runtime for each sample was less than 15 min, in which all eight monoamines and their metabolites (MHPG, NA, A, DOPAC, 5-HIAA, DA, HVA and 5-HT) were determined in this respective order. MHPG/NA, DOPAC/DA, HVA/DA and 5-HIAA/5-HT ratios were calculated as indicators for noradrenergic, dopaminergic and serotonergic metabolic turnover, respectively, whereas the HVA/5-HIAA ratio was included as an indicator for serotonergic inhibition of dopaminergic transmission.

### 2.5 Statistical analysis

Normality of distribution was assessed by means of Shapiro-Wilkinson tests, which indicated that H/M, monoaminergic and biomarker data were all abnormally distributed in all groups. For this reason, non-parametric Spearman’s correlations was performed to investigate correlation strength of H/M values with CSF, serum and plasma monoamines, as well as with CSF biomarker levels, in the entire population and in the population free from psychotropic medication. In addition, non-parametric Mann-Whitney U tests were applied to detect potential alterations in monoaminergic and CSF biomarker levels between different sexes and between patients taking versus not taking different types of psychotropic medication. Linear regression was performed to investigate the effect of age on H/M values and monoamine levels. The significance level was set at 0.05. Correlation plots were created in RStudio for R (2023, PBC, Boston, MA, USA).

## 3 Results

### 3.1 Demographic information

All demographic information is summarized in Table 1. In total, 44 possible DLB patients were included. Median age at the time of the MIBG scan and LP sampling was 75.6 years and there was a 23:21 male-to-female ratio. The median time between MIBG scanning and LP sampling was 4.1 weeks (IQR: 2.1–13.1 weeks). 35 out of 44 patients had a positive MIBG status. Of all patients, 86.4 % was taking psychotropic medication. Linear regression analysis revealed no significant effects of age on H/M values or monoaminergic levels. With regard to biological sex, there was a significant effect on CSF A (R^2^ = 0.142, F(1,36) = 5.944, *P* = 0.020, CSF DOPAC (R^2^ = 0.106, F(1,42) = 4.984, *P* = 0.031), serum NA (R^2^ = 0.207, F(1,30) = 7.845, *P* = 0.009) and serum 5-HT levels (R^2^ = 0.150, F(1,27) = 4.767, *P* = 0.038). Indeed, a Mann Whitney *U* test confirmed that serum NA (*U* = 51.0, *P* = 0.004), serum 5-HT (*U* = 54.0, *P* = 0.026) and serum 5-HIAA/5-HT levels (*U* = 150.0, *P* = 0.003) were significantly higher in male than in female participants (Supplementary file 1).

**Table 1:** Demographic information and medication use.

**Parameter**	**DLB** **(n = 44)**
**Age at MIBG scan (y)**	75.6 (71.8–81.0) (n = 44)
**Age at LP sampling (y)**	75.6 (71.7–80.1) (n = 44)
**Sex** **(Male/Female)**	23/21
**MIBG status** **(Positive/Negative)**	35/9
**Psychotropic medication** **(No/Yes)**	6/38
**Antidepressants** **(No/Yes)**	18/26
**Antipsychotics** **(No/Yes)**	31/13
**Antiepileptics** **(No/Yes)**	42/2
**Anxiolytics & hypnotics** **(No/Yes)**	25/19
**Cholinesterase inhibitors** **(No/Yes)**	31/13
**Antidementia medication** **(No/Yes)**	30/14
**Analgesics** **(No/Yes)**	43/1
**Anti-Parkinsonian medication** **(No/Yes)**	31/13

Age is depicted as median with interquartile ranges between parentheses. Abbreviations: DLB = dementia with Lewy bodies, LP = lumbar puncture, MIBG = metaiodobenzylguanidine.

### 3.2 Correlation between H/M ratio and monoaminergic levels

**Fig. 1:**
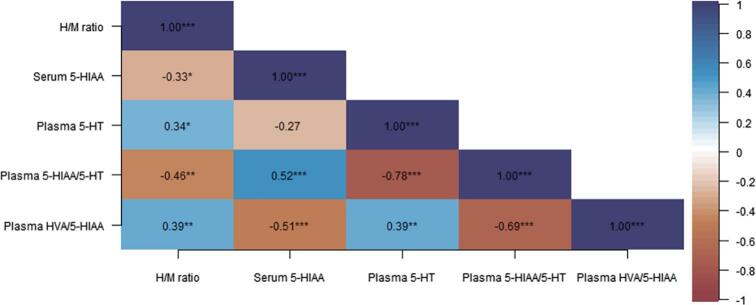
Correlations between H/M ratio, blood 5-HIAA, 5-HT levels and 5-HIAA/5-HT and HVA/5-HIAA ratios. Correlation strength is visualized by color shade and correlation coefficients. Significance values are classified as P ≤ 0.05, P ≤ 0.01 and P ≤ 0.001 and are indicated by one, two or three asterisk symbols, respectively. Abbreviations: H/M = heart-to-mediastinum, 5-HIAA = 5-hydroxyindoleacetic acid, DA = dopamine, HVA = homovanillic acid, 5-HT = 5-hydroxytryptamine (serotonin).

We found statistically significant correlations between the H/M ratio and serum 5-HIAA (*r_s_*(39) = -0.330, *P* = 0.035, two-tailed), plasma 5-HT (*r_s_*(36) = 0.342, *P* = 0.036, two-tailed), plasma 5-HIAA/5-HT (*r_s_*(36) = -0.464, *P* = 0.003, two-tailed) and plasma HVA/5-HIAA (*r_s_*(42) = 0.387, *P* = 0.009, two-tailed), but no further correlations with the noradrenergic system (Fig. 1, Table 2).

**Table 2:** Correlations of the H/M ratio with CSF, plasma and serum monoaminergic levels.

**Parameter**		**DLB** **(n = 44)**	**Correlation to** **H/M ratio**
**H/M ratio**		1.360 (1.100–1.653) (n = 44)^a,b,c,d^	/
**MHPG** **(ng/mL)**	CSF	16.229 (9.922–20.687) (n = 44)	ρ = 0.107 *P* > 0.05
	Plasma	17.856 (6.487–30.549) (n = 42)	ρ = 0.063 *P* > 0.05
	Serum	21.392 (9.132–37.025) (n = 42)	ρ = -0.098 *P* > 0.05
**NA** **(ng/mL)**	CSF	0.707 (0.487–1.701) (n = 33)	ρ = -0.060 *P* > 0.05
	Plasma	1.559 (0.620–2.900) (n = 38)	ρ = -0.058 *P* > 0.05
	Serum	0.901 (0.381–2.578) (n = 32)	ρ = -0.029 *P* > 0.05
**A** **(ng/mL)**	CSF	2.715 (2.306–3.398) (n = 38)	ρ = -0.060 *P* > 0.05
	Plasma	11.031 (8.028–17.050) (n = 40)	ρ = 0.148 *P* > 0.05
	Serum	4.234 (2.315–9.877) (n = 32)	ρ = 0.110 *P* > 0.05
**DOPAC** **(ng/mL)**	CSF	0.910 (0.606–2.333) (n = 44)	ρ = 0.191 *P* > 0.05
	Plasma	2.227 (1.148–4.946) (n = 41)	ρ = 0.152 *P* > 0.05
	Serum	3.704 (2.429–7.431) (n = 39)	ρ = -0.167 *P* > 0.05
**5-HIAA** **(ng/mL)**	CSF	17.821 (12.178–24.745) (n = 44)	ρ = 0.068 *P* > 0.05
	Plasma	14.407 (5.028–21.292) (n = 44)	ρ = -0.221 *P* > 0.05
	Serum	15.402 (4.721–21.982) (n = 41)^a^	**ρ = -0.330** ***P* = 0.035**
**DA** **(ng/mL)**	CSF	1.353 (1.058–1.689) (n = 44)	ρ = -0.081 *P* > 0.05
	Plasma	2.750 (0.983–6.032) (n = 44)	ρ = -0.086 *P* > 0.05
	Serum	0.674 (0.240–0.946) (n = 32)	ρ = 0.088 *P* > 0.05
**HVA** **(ng/mL)**	CSF	37.143 (23.521–52.599) (n = 44)	ρ = 0.078 *P* > 0.05
	Plasma	12.892 (6.920–24.456) (n = 44)	ρ = -0.049 *P* > 0.05
	Serum	12.372 (7.023–28.055) (n = 35)	ρ = 0.063 *P* > 0.05
**5-HT** **(ng/mL)**	CSF	0.382 (0.318–0.571) (n = 41)	ρ = 0.126 *P* > 0.05
	Plasma	3.150 (1.450–8.070) (n = 38)^b^	**ρ = 0.342** ***P* = 0.036**
	Serum	23.584 (6.094–58.679) (n = 29)	ρ = -0.132 *P* > 0.05
**MHPG/NA**	CSF	27.007 (8.887–35.568) (n = 33)	ρ = 0.087 *P* > 0.05
	Plasma	9.711 (4.486–35.417) (n = 35)	ρ = 0.062 *P* > 0.05
	Serum	17.763 (8.281–76.106) (n = 37)	ρ = 0.017 *P* > 0.05
**DOPAC/DA**	CSF	0.740 (0.416–3.858) (n = 44)	ρ = 0.124 *P* > 0.05
	Plasma	1.276 (0.257–4.500) (n = 41)	ρ = 0.030 *P* > 0.05
	Serum	9.645 (4.652–27.080) (n = 28)	ρ = -0.358 *P* > 0.05
**HVA/DA**	CSF	26.421 (17.068–49.538) (n = 44)	ρ = 0.052 *P* > 0.05
	Plasma	7.581 (1.752–20.350) (n = 44)	ρ = 0.064 *P* > 0.05
	Serum	34.584 (14.440–155.789) (n = 23)	ρ = -0.203 *P* > 0.05
**5-HIAA/5-HT**	CSF	38.716 (25.559–71.881) (n = 41)	ρ = -0.091 *P* > 0.05
	Plasma	4.292 (0.753–47.355) (n = 38)^c^	**ρ = -0.464** ***P* = 0.003**
	Serum	0.388 (0.220–2.510) (n = 27)	ρ = 0.044 ρ = 9.974
**HVA/5-HIAA**	CSF	2.084 (1.597–2.796) (n = 44)	ρ = -0.070 *P* > 0.05
	Plasma	0.933 (0.630–1.531) (n = 44)^d^	**ρ = 0.387** ***P* = 0.009**
	Serum	0.760 (0.420–1.617) (n = 32)	ρ = 0.295 *P* > 0.05

We repeated the analysis in patients with a positive MIBG status (n = 35), and found highly similar results. There statistically significant correlations between the H/M ratio and serum A (*r_s_*(25) = 0.410, *P* = 0.034, two-tailed), serum 5-HIAA (*r_s_*(31) = -0.430, *P* = 0.012, two-tailed), plasma 5-HT (*r_s_*(29) = 0.508, *P* = 0.004, two-tailed), plasma 5-HIAA/5-HT (*r_s_*(29) = -0.579, *P* < 0.001, two-tailed) and plasma HVA/5-HIAA (*r_s_*(33) = 0.423, *P* = 0.011, two-tailed) (Supplementary file 2).

In addition, there was significant correspondence between CSF, serum and plasma monoaminergic compounds. CSF-serum MHPG (*r_s_*(40) = 0.478, *P* = 0.001, two-tailed), CSF-serum DOPAC (*r_s_*(37) = 0.490, *P* = 0.002, two-tailed), CSF-serum HVA (*r_s_*(33) = 0.400, *P* = 0.017), CSF-plasma MHPG (*r_s_*(36) = 0.391, *P* = 0.015), CSF plasma NA (*r_s_*(29) = 0.524, *P* = 0.002), CSF-plasma DOPAC (*r_s_*(39) = 0.471, *P* = 0.002), CSF plasma HVA (*r_s_*(42) = 0.397, *P* = 0.008) and CSF-plasma MHPG/NA (*r_s_*(27) = 0.399, *P* = 0.032) were significantly correlated. We also noted significant correlations between serum and plasma MHPG (*r_s_*(35) = 0.689, *P* < 0.001, two-tailed), DOPAC (*r_s_*(34) = 0.480, *P* = 0.003, two-tailed), 5-HIAA (*r_s_*(37) = 0.372, *P* = 0.017, two-tailed), HVA (*r_s_*(33) = 0.648, *P* < 0.001, two-tailed), 5-HT (*r_s_*(21) = 0.424, *P* = 0.044, two-tailed), MHPG/NA (*r_s_*(28) = 0.700, *P* < 0.001, two-tailed), 5-HIAA/5-HT (*r_s_*(19) = 0.643, *P* = 0.002, two-tailed) and HVA/5-HIAA (*r_s_*(30) = 0.455, *P* = 0.009, two-tailed).

Data are presented as median with interquartile ranges between brackets. Spearman correlations tests were performed to correlate monoaminergic levels to the H/M ratio in possible DLB patients. Statistical outcome is listed in the rightmost column. Significance values are classified as *P* ≤ 0.05 and are indicated by a superscript letter. The letters a, b, c and d denote significant correlations between the H/M ratio and serum 5-HIAA^a^, the H/M ratio and plasma 5-HT^b^_,_ the H/M ratio and plasma 5-HIAA/5-HT^c^ and between the H/M ratio and plasma HVA/5-HIAA^d^. Abbreviations: CSF = cerebrospinal fluid, DLB = dementia with Lewy bodies, H/M = heart-to-mediastinum, MHPG = 3-methoxy-4-hydroxyphenylglycol, NA = noradrenaline, A = adrenaline, DOPAC = 3,4-dihydroxyphenylacetic acid, 5-HIAA = 5-hydroxyindoleacetic acid, DA = dopamine, HVA = homovanillic acid, 5-HT = 5-hydroxytryptamine (serotonin).

### 3.3 Effect of psychotropic medication and sex

The use of psychotropic medication significantly affected monoaminergic levels (Supplementary files 3–10). Patients taking psychotropic medication had significantly elevated CSF 5-HIAA (*U* = 179.5, *P* = 0.025), CSF HVA (*U* = 187.0, *P* = 0.013), CSF HVA/DA (*U* = 189.0, *P* = 0.010), CSF 5-HIAA/5-HT (*U* = 146.0, *P* = 0.026), serum HVA (*U* = 126.0, *P* = 0.016), plasma 5-HIAA (*U* = 184.0, *P* = 0.017) and plasma HVA (*U* = 191.0, *P* = 0.008) levels compared to those who did not (Supplementary file 3). Specifically, antidepressant use led to significantly higher CSF HVA (*U* = 177.0, *P* = 0.040), CSF HVA/DA (*U* = 317.0, *P* = 0.048), serum NA (*U* = 177.0, *P* = 0.040), serum HVA (*U* = 235.0, *P* = 0.003), plasma 5-HIAA (*U* = 318.0, *P* = 0.045) and plasma HVA levels (*U* = 324.0, *P* = 0.032), but lower serum MHPG/NA (*U* = 62.0, *P* = 0.018) and plasma A levels (*U* = 116.0, *P* = 0.046) (Supplementary file 4). There was a positive association between antipsychotic use and serum A levels (*U* = 184.0, *P* = 0.034) (Supplementary file 5). Patients receiving anxiolytic treatment had significantly higher CSF 5-HIAA/5-HT (*U* = 285.0, *P* = 0.040), serum 5-HT (*U* = 55.0, *P* = 0.037), serum DOPAC/DA (*U* = 154.0, *P* = 0.007) and serum 5-HIAA/5-HT levels (*U* = 144.0, *P* = 0.006) (Supplementary file 6). On the other hand, CSF 5-HIAA levels were significantly higher (*U* = 298.0, *P* = 0.013), and serum DOPAC/DA ratios significantly lower in patients taking cholinesterase inhibitors (*U* = 40.0, *P* = 0.042) (Supplementary file 7). Similarly, we noted significantly lower serum DOPAC/DA ratios in patients receiving overall antidementia medication (*U* = 35.0, *P* = 0.013) (Supplementary file 8). Surprisingly, serum DA (*U* = 48.0, *P* = 0.012) were significantly lower in patients taking antiparkinsonian (DA-elevating) medication, whereas plasma MHPG levels were significantly higher (*U* = 204.0, *P* = 0.034) (Supplementary file 9). Analgetic use could not be compared since only one patient received analgetic treatment. There was no significant correlation of any type of psychotropic medication with H/M values.

We repeated the correlation analysis in the population free from psychotropic medication (n = 6) and free from antidepressant treatment (n = 18). In the population free from psychotropic medication, we found no significant correlations of the H/M ratio with any of the monoaminergic or biomarker levels. In the cohort not taking antidepressant treatment, we found that the H/M ratio was positively correlated to CSF HVA levels (*r_s_*(18) = 0.474, *P* = 0.047), serum HVA/5-HIAA (*r_s_*(13) = 0.787, *P* = 0.001) and plasma HVA/5-HIAA ratios (*r_s_*(18) = 0.621, *P* = 0.006), whereas plasma 5-HIAA/5-HT ratios were negatively correlated (*r_s_*(15) = -0.565, *P* = 0.028) (Supplementary file 10).

Considering the effect of biological sex on CSF A, CSF DOPAC, serum NA and serum 5-HT levels, correlations between these monoaminergic levels with the H/M ratio were repeated while stratified by sex. However, this did not reveal significant correlations (data not shown).

## 4 Discussion

Since the literature demonstrates parallel changes in noradrenergic denervation and NA levels in multiple brain regions, CSF and serum in DLB [3,13,15], we hypothesized to observe a link between peripheral noradrenergic denervation and circulating central and peripheral noradrenergic levels. However, our results do not support this hypothesis. Instead, we found a significant correlation between noradrenergic denervation, as measured by the H/M ratio, with the serotonergic system. Upon repeating the analysis in a cohort with positive MIBG status, highly similar correlations were found, further supporting our results. In the population free from psychotropic medication, which may affect monoaminergic levels, no association was found between the H/M ratio and any of the monoaminergic compounds. Finally, there were significant correlations between CSF and serum/plasma levels of monoaminergic metabolites, more specifically between CSF-serum MHPG, DOPAC, HVA, CSF-plasma MHPG, NA, DOPAC, MHPG/NA, DOPAC/DA and serum-plasma MHPG, DOPAC, 5-HIAA, HVA, 5-HT, MHPG/NA, 5-HIAA/5-HT and HVA/5-HIAA.

### 4.1 H/M-determined noradrenergic uptake is inversely correlated to serotonergic activity

As mentioned earlier, we demonstrated a significant link between the H/M ratio and the serotonergic system. H/M ratio was negatively correlated with serum 5-HIAA and plasma 5-HIAA/5-HT, whereas it was positively correlated with plasma 5-HT and plasma HVA/5-HIAA, indicating that decreased H/M ratios are associated with increased serotonergic turnover. This could be a result of an inhibitory effect of 5-HT on MIBG reuptake by NA transporters, as observed in neuroblastoma cell lines and patients with neuroblastoma [24,25]. If this is the case, decreased H/M values would not necessarily signify a loss of noradrenergic innervation in DLB patients, but could suggest a similar shift towards increased serotonin in DLB patients. This could be a result of increased density of NA, DA and 5-HT transporters inhibiting MIBG reuptake, although the underlying mechanism is not yet fully understood. A study by Blom et al. [26] suggests that competitive uptake of MIBG by serotonin transporters might be at the root of this phenomenon. These findings are in line with those of Glowniak et al. [27], namely that inhibition of DA and 5-HT transporters strongly inhibit non-specific or non-neuronal MIBG uptake. Another study reports the uptake of MIBG, in addition to NA and 5-HT, by platelets, indicating that MIBG and 5-HT might have similar selectivity for non-neuronal uptake [28].

In accordance with these studies, we might expect statistically significant correlations between noradrenergic and dopaminergic levels with MIBG reuptake, which we did not observe. Alternatively, the use of antidepressants, more specifically selective serotonergic/noradrenergic reuptake inhibitors (SSRI/SNRI), tricyclic or atypical antidepressants, could offer an explanation for this serotonergic link [29,30]. For this reason, we also repeated the correlation tests in a population free from antidepressant treatment (n = 18), and found that CSF HVA levels, serum HVA/5-HIAA and plasma HVA/5-HIAA ratios were positively correlated to the H/M ratio, whereas plasma 5-HIAA/5-HT ratios were inversely correlated. While these correlations slightly differ from those in the total study population, we also noted similar correlations. Hence, we cannot attribute our findings merely to the use of serotonin/noradrenergic reuptake-inhibiting therapies, but rather have to see this as part of a more complex underlying interaction.

To our knowledge, this is the first study to investigate the relationship between blood and CSF monoaminergic neurotransmitters and H/M values of MIBG scans in DLB patients. However, several other studies reported links between the monoaminergic neurotransmission and MIBG uptake in patients with parkinsonism and PD(D). For example, Murakami et al. [31] and Shimasaki et al. [32] both reported significant positive correlations between CSF 5-HIAA levels and the delayed H/M ratio in drug-naïve PD(D) patients. Contrarily, we observed significant negative correlations between the delayed H/M ratio and serum and plasma 5-HIAA in DLB patients. However, the use of psychotropic medication might also explain the discrepancy between our studies: except for dopaminergic medication for PD(D) treatment, no other types of psychotropic medication were used, as opposed to our study. A case report study by Muraoka et al. [33], describes the occurrence of parkinsonian features and reduced MIBG uptake upon treatment with an SSRI/SNRI, indicating that increased serotonergic turnover might indeed be linked to reduced H/M ratios. Another DLB autopsy case, however, also demonstrates decreased CSF 5-HIAA and HVA alongside a decreased H/M ratio [34].

There were significant correlations between monoaminergic levels in CSF and serum/plasma, more specifically between CSF-serum MHPG, DOPAC, HVA, CSF-plasma MHPG, NA, DOPAC, HVA and MHPG/NA. This indicates that CSF levels of monoaminergic metabolites could also be reflected in blood, and corroborates earlier findings indicating passive diffusion of monoaminergic metabolites over the CSF-blood barrier [35]. On the other hand, 5-HIAA and 5-HT might be transported across the CSF-blood barrier actively rather than passively [36,37], which could explain the differences in H/M ratio and serotonergic correlations between our study and that of Murakami et al. [31] and Shimasaki et al. [32]. Between serum and plasma, MHPG, DOPAC, 5-HIAA, HVA, 5-HT levels and MHPG/NA, 5-HIAA/5-HT and HVA/5-HIAA ratios were all moderately to strongly correlated. Nevertheless, we did not detect similar significant correlations of the H/M ratio with 5-HIAA, 5-HT levels, 5-HIAA/5-HT and HVA/5-HIAA ratios in both serum and plasma. This discrepancy could be a consequence of the storage of 5-HT in blood platelets. While platelet coagulation in serum leads to platelet activation and subsequent 5-HT release, anticoagulants used in plasma collection prevent this, thus leading to generally lower plasma 5-HT levels [38,39].

### 4.2 The effect of demographic factors on H/M, monoaminergic and biomarker levels

Several demographic factors affected H/M values, as well as monoaminergic and CSF biomarker levels. For example, serum 5-HT was significantly higher in male compared to female subjects. The serotonergic system has been described as subject to hormonal changes [40], which could explain this finding. In addition, this might be a result of antidepressant treatment, which is known to inhibit ^123^I-MIBG reuptake [30]. However, we found no difference in antidepressant use between males and females. We found no association between the H/M ratio and sex or age in our study, in contrast to others who reported a significant decrease in H/M values with ageing [41]. Psychotropic treatment in most patients in this study could have caused this discrepancy, since no patients in the study of Sakata et al. [41] received medication for the autonomous nervous system and Slaets et al. [7] corrected for antidepressant treatment. However, we found no correlation between H/M values with any type of psychotropic medication.

### 4.3 Study limitations

There are several limitations with regards to this study. The population free from psychotropic medication was very small (n = 6), which undoubtedly reduced statistical power. Moreover, considering only two patients received antiepileptic medication, further analyses between antiepileptics and monoaminergic levels were excluded. Furthermore, there was no information available regarding disease duration or severity, which may substantially affect biomarker status such as the H/M ratio or monoaminergic concentrations. In addition, the patients in this study received a DLB diagnosis based on the previous diagnostic criteria from 2005 [22]. Based on the more recent 2017 criteria [23], and a predefined H/M cut-off ratio of 1.68 to indicate ^123^I-MIBG-positivity [5], 35 out of 44 patients would receive a probable DLB diagnosis instead of a possible DLB diagnosis. However, we decided not to classify our study population into possible and probable DLB patients, since a universally approved ^123^I-MIBG-positivity cut-off value has not yet been defined. Instead, we repeated the analysis in the cohort with a positive MIBG status. As this is a correlation analysis in DLB subjects, no control or AD group was included, although this could have shed a light on the correlation between the H/M ratio and monoaminergic levels in DLB versus AD or non-demented individuals. In addition, we did not record the presence of cardiovascular autonomic failure, although this condition has also been linked to cardiac sympathetic denervation and thus abnormal MIBG-uptake [42]. However, cardiac sympathetic denervation also occurs separately from autonomic failure [43], and the sensitivity of MIBG-scintigraphy as a diagnostic measure for autonomic failure, has not yet been unanimously proven [44,45].

### 4.4 Conclusion & future perspectives

This study found a significant correlation between peripheral sympathetic (noradrenergic) denervation and serotonergic neurotransmission. The underlying mechanism linking this primarily noradrenergic denervation with increased serotonergic levels certainly requires more investigation. The inclusion of a control group, PDD, mixed DLB-AD and/or AD patients in future studies is recommended to investigate disease-specific differences between sympathetic (noradrenergic) denervation and monoamine neurotransmitter systems. Ideally, researchers should also attempt to include more patients free of psychotropic medication. This will probably remain a clinical and ethical challenge, given that many dementia patients receive psychotropic medication to manage their behavioral disturbances. Ultimately, unraveling the link of serotonergic (and by extension monoaminergic) neurotransmission with DLB-related pathology could reveal disease-specific monoaminergic alterations and might present fluid CSF or blood monoaminergic levels as valuable additional diagnostic biomarkers in DLB. In conclusion, this study offers an intriguing insight in the relationship between peripheral noradrenergic reuptake and monoaminergic neurotransmission and could serve as a starting point to further explore this link.

## CRediT authorship contribution statement

**Annelies Heylen:** Writing – original draft, Investigation, Formal analysis. **Yannick Vermeiren:** Writing – review & editing, Methodology, Funding acquisition, Conceptualization. **Sebastiaan Engelborghs:** Writing – review & editing, Resources. **Frank Van Acker:** Writing – review & editing, Methodology. **Peter Paul De Deyn:** Writing – review & editing, Supervision, Resources, Funding acquisition, Conceptualization. **Debby Van Dam:** Writing – review & editing, Supervision, Resources, Funding acquisition, Conceptualization.

## Declaration of competing interest

The authors declare that they have no known competing financial interests or personal relationships that could have appeared to influence the work reported in this paper.
