# Supplementary material for: Correlation analyses between MIBG myocardial scintigraphy and monoamine levels in dementia with Lewy bodies show potential link with the serotonergic system
Source: Clin Park Relat Disord. 2025 May 12;12:100346. doi: 10.1016/j.prdoa.2025.100346 (PMC12143658; doi:10.1016/j.prdoa.2025.100346)
Supplement: Supplementary Data 1 [file mmc1.docx]

**Correlation analyses between MIBG myocardial scintigraphy and monoamine levels in dementia with Lewy bodies show potential link with the serotonergic system**

Heylen Annelies *^1^*, Vermeiren Yannick *^2,3^*, Engelborghs Sebastiaan *^1,4,5^*, Van Acker, Frank *^6^*, De Deyn Peter Paul *^1,7^*, Van Dam Debby *^1,7^*

*^1^ Laboratory of Neurochemistry and Behaviour, Experimental Neurobiology unit, Department of Biomedical Sciences, University of Antwerp, Antwerp, Belgium.*

*^2^ Division of Human Nutrition and Health, Chair Group of Nutritional Biology, Wageningen University & Research (WUR), Wageningen, the Netherlands*

*^3^ Faculty of Medicine & Health Sciences, Translational Neurosciences, University of Antwerp, Antwerp, Belgium*

*^4^ Department of Neurology, Universitair Ziekenhuis Brussel (UZ Brussel), Brussels, Belgium.*

*^5^ Neuroprotection and Neuromodulation (NEUR) Research Group, Center for Neurosciences, Vrije Universiteit Brussel, Brussels, Belgium.*

*^6^* Department of Nuclear Medicine, Ziekenhuis Aan de Stroom (ZAS) Middelheim, Antwerp, Belgium.

*^7^ Department of Neurology and Alzheimer Research Center, University of Groningen and University Medical Center Groningen, Groningen, the Netherlands.*

**Corresponding author**

Prof. dr. Debby Van Dam

Debby.vandam@uantwerpen.be

[d.c.j.van.dam@umcg.nl](mailto:d.c.j.van.dam@umcg.nl)

| **Supplementary Table 1. Significant sex differences in serum monoaminergic levels of DLB patients** | | | | |
| --- | --- | --- | --- | --- |
| **Parameter** |  | **Male**  **(n = 23)** | **Female**  **(n = 21)** | **Mann-Whitney U statistics** |
| **NA**  **(ng/mL)** | Serum | 1.507  (0.731-3.231)  (n = 18) | 0.450  (0.133-0.983)  (n = 14) | *U* = 51.0  *P* = 0.004 |
| **5-HT**  **(ng/mL)** | Serum | 44.167  (17.984-81.961)  (n = 15) | 11.400  (3.001-40.004)  (n = 14) | *U* = 54.0  *P* = 0.026 |
| **5-HIAA/5-HT** | Serum | 0.296  (0.168-0.369)  (n = 14) | 1.113  (0.447-10.284)  (n = 13) | *U* = 150.0  *P* = 0.003 |
| Data are presented as median with interquartile ranges between brackets. Mann-Whitney U tests were performed to discern sex differences in monoaminergic levels of DLB patients. Statistical outcome is listed in the rightmost column. Abbreviations: DLB = dementia with Lewy bodies, NA = noradrenaline, 5-HIAA = 5-hydroxyindoleacetic acid, 5-HT = 5-hydroxytryptamine (serotonin). | | | | |

| **Supplementary Table 2. Significant correlations of the H/M ratio with CSF, serum and plasma monoaminergic levels of DLB patients with positive MIBG status.** | | | |
| --- | --- | --- | --- |
| **Parameter** |  | **DLB**  **(n = 35)** | **Correlation to**  **H/M ratio** |
| **H/M ratio** |  | 1.240  (1.070-1.500)  (n = 35) | / |
| **A**  **(ng/mL)** | Serum | 5.036  (2.758-9.737)  (n = 27) | ρ = 0.410  *P* = 0.034 |
| **5-HIAA**  **(ng/mL)** | Serum | 16.396  (4.368-22.100)  (n = 33) | ρ = -0.430  *P* = 0.012 |
| **5-HT**  **(ng/mL)** | Plasma | 3.050  (1.450-7.790)  (n = 31) | ρ = 0.508  *P* = 0.004 |
| **5-HIAA/5-HT** | Plasma | 4.890  (1.013-51.768)  (n = 31) | ρ = -0.579  *P* < 0.001 |
| **HVA/5-HIAA** | Plasma | 0.889  (0.551-1.421)  (n = 35) | ρ = 0.423  *P* = 0.011 |
| Data are presented as median with interquartile ranges between brackets. Spearman correlations tests were performed to correlate monoaminergic levels to the H/M ratio in DLB patients with positive MIBG status. Statistical outcome is listed in the rightmost column. Abbreviations: CSF = cerebrospinal fluid, DLB = dementia with Lewy bodies, H/M = heart-to-mediastinum, MIBG = metaiodobenzylguanidine, A = adrenaline, 5-HIAA = 5-hydroxyindoleacetic acid, HVA = homovanillic acid, 5-HT = 5-hydroxytryptamine (serotonin). | | | |

| **Supplementary Table 3. Significant differences in CSF, serum and plasma monoaminergic levels of DLB patients taking vs not taking psychotropic medication** | | | | |
| --- | --- | --- | --- | --- |
| **Parameter** |  | **Psychotropic medication**  **(n = 38)** | **No psychotropic medication**  **(n = 6)** | **Mann-Whitney U statistics** |
| **5-HIAA**  **(ng/mL)** | CSF | 13.277  (18.866-25.232)  (n = 38) | 12.859  (6.464-14.874)  (n = 6) | *U* = 179.5  *P* = 0.025 |
|  | Plasma | 14.854  (7.553-49.574)  (n = 38) | 4.168  (3.262-11.997)  (n = 6) | *U* = 184.0  *P* = 0.017 |
| **HVA**  **(ng/mL)** | CSF | 37.996  (29.115-56.320)  (n = 38) | 15.031  (11.455-33.571)  (n = 6) | *U* = 126.0  *P* = 0.013 |
|  | Serum | 15.826  (8.177-32.945)  (n = 30) | 5.987  (4.572-9.065)  (n = 5) | *U* = 126.0  *P* = 0.016 |
|  | Plasma | 16.538  (8.261-24.718)  (n = 38) | 3.102  (5.438-10.923)  (n = 6) | *U* = 191.0  *P* = 0.008 |
| **HVA/DA** | CSF | 27.822  (21.086-50.994)  (n = 38) | 9.498  (7.697-23.910)  (n = 6) | *U* = 189.0  *P* = 0.010 |
| **5-HIAA/5-HT** | CSF | 43.170  (26.822-73.401)  (n = 36) | 16.523  (13.364-38.237)  (n = 5) | *U* = 146.0  *P* = 0.026 |
| Data are presented as median with interquartile ranges between brackets. Mann-Whitney U tests were performed to discern differences in monoaminergic levels of DLB patients taking vs not taking psychotropic medication. Statistical outcome is listed in the rightmost column. Abbreviations: DLB = dementia with Lewy bodies, CSF = cerebrospinal fluid, DA = dopamine, HVA = homovanillic acid, 5-HIAA = 5-hydroxyindoleacetic acid, 5-HT = 5-hydroxytryptamine (serotonin). | | | | |

| **Supplementary Table 4. Significant differences in CSF, serum and plasma monoaminergic levels of DLB patients taking vs not taking antidepressants** | | | | |
| --- | --- | --- | --- | --- |
| **Parameter** |  | **Antidepressants**  **(n = 26)** | **No antidepressants**  **(n = 18)** | **Mann-Whitney U statistics** |
| **NA**  **(ng/mL)** | Serum | 1.281  (0.476-2.868)  (n = 19) | 0.598  (0.135-1.067)  (n = 13) | *U* = 177.0  *P* = 0.040 |
| **A**  **(ng/mL)** | Plasma | 10.255  (7.640-12.526)  (n = 25) | 15.722  (8.610-25.342)  (n = 15) | *U* = 116.0  *P* = 0.046 |
| **5-HIAA**  **(ng/mL)** | Plasma | 16.035  (7.745-54.626)  (n = 26) | 7.613  (4.012-17.658)  (n = 18) | *U* = 318.0  *P* = 0.045 |
| **HVA**  **(ng/mL)** | CSF | 40.374  (33.878-72.801)  (n = 26) | 28.560  (17.250-44.649)  (n = 18) | *U* = 177.0  *P* = 0.040 |
|  | Serum | 19.235  (9.163-47.462)  (n = 21) | 8.311  (5.706-11.339)  (n = 14) | *U* = 235.0  *P* = 0.003 |
|  | Plasma | 18.598  (9.030-25.875)  (n = 26) | 7.674  (5.428-23.028)  (n = 18) | *U* = 324.0  *P* = 0.032 |
| **MHPG/NA** | Serum | 10.023  (7.315-24.711)  (n = 19) | 51.881  (15.170-231.762)  (n = 13) | *U* = 62.0  *P* = 0.018 |
| **HVA/DA** | CSF | 32.060  (23.752-50.994)  (n = 26) | 20.734  (11.247-39.341)  (n = 18) | *U* = 317.0  *P* = 0.048 |
| Data are presented as median with interquartile ranges between brackets. Mann-Whitney U tests were performed to discern differences in monoaminergic levels of DLB patients taking vs not taking antidepressants. Statistical outcome is listed in the rightmost column. Abbreviations: DLB = dementia with Lewy bodies, CSF = cerebrospinal fluid, NA = noradrenaline, A = adrenaline, HVA = homovanillic acid, MHPG = 3-methoxy-4-hydroxyphenylglycol, DA = dopamine, 5-HIAA = 5-hydroxyindoleacetic acid, 5-HT = 5-hydroxytryptamine (serotonin). | | | | |

| **Supplementary Table 5. Significant differences in serum monoaminergic levels of DLB patients taking vs not taking antipsychotics** | | | | |
| --- | --- | --- | --- | --- |
| **Parameter** |  | **Antipsychotics**  **(n = 13)** | **No antipsychotics**  **(n = 31)** | **Mann-Whitney U statistics** |
| **A** | Serum | 9.737  (3.869-12.677)  (n = 11) | 3.041  (1.923-6.275)  (n = 23) | *U* = 184.0  *P* = 0.034 |
| Data are presented as median with interquartile ranges between brackets. Mann-Whitney U tests were performed to discern differences in monoaminergic levels of DLB patients taking vs not taking antipsychotics. Statistical outcome is listed in the rightmost column. Abbreviations: A = adrenaline, DLB = dementia with Lewy bodies. | | | | |

| **Supplementary Table 6. Significant differences in CSF and serum monoaminergic levels of DLB patients taking vs not taking anxiolytics** | | | | |
| --- | --- | --- | --- | --- |
| **Parameter** |  | **Anxiolytics**  **(n = 19)** | **No anxiolytics**  **(n = 25)** | **Mann-Whitney U statistics** |
| **5-HT**  **(ng/mL)** | Serum | 15.312  (3.124-34.813)  (n = 12) | 51.990  (14.148-74.542)  (n = 17) | *U* = 55.0  *P* = 0.037 |
| **DOPAC/DA** | Serum | 25.490  (7.922-125.109)  (n = 12) | 5.786  (3.640-13.612)  (n = 16) | *U* = 154.0  *P* = 0.007 |
| **5-HIAA/5-HT** | CSF | 53.289  (31.881-82.352)  (n = 18) | 34.695  (22.438-43.361)  (n = 23) | *U* = 285.0  *P* = 0.040 |
|  | Serum | 1.140  (0.388-12.253)  (n = 11) | 0.296  (0.175-0.477)  (n = 16) | *U* = 144.0  *P* = 0.006 |
| Data are presented as median with interquartile ranges between brackets. Mann-Whitney U tests were performed to discern differences in monoaminergic levels of DLB patients taking vs not taking anxiolytics. Statistical outcome is listed in the rightmost column. Abbreviations: DLB = dementia with Lewy bodies, CSF = cerebrospinal fluid, NA = noradrenaline, A = adrenaline, DA = dopamine, DOPAC = 3,4-dihydroxyphenylacetic acid, 5-HIAA = 5-hydroxyindoleacetic acid, 5-HT = 5-hydroxytryptamine (serotonin). | | | | |

| **Supplementary Table 7. Significant differences in CSF and serum monoaminergic levels of DLB patients taking vs not taking cholinesterase inhibitors** | | | | |
| --- | --- | --- | --- | --- |
| **Parameter** |  | **Cholinesterase inhibitors**  **(n = 13)** | **No cholinesterase inhibitors**  **(n = 31)** | **Mann-Whitney U statistics** |
| **5-HIAA**  **(ng/mL)** | CSF | 24.950  (16.393-30.906)  (n = 13) | 14.916  (10.929-21.365)  (n = 31) | *U* = 298.0  *P* = 0.013 |
| **DOPAC/DA** | Serum | 4.993  (2.665-13.612)  (n = 8) | 14.597  (5.189-66.380)  (n = 20) | *U* = 40.0  *P* = 0.042 |
| Data are presented as median with interquartile ranges between brackets. Mann-Whitney U tests were performed to discern differences in monoaminergic levels of DLB patients taking vs not taking cholinesterase inhibitors. Statistical outcome is listed in the rightmost column. Abbreviations: DLB = dementia with Lewy bodies, CSF = cerebrospinal fluid, DA = dopamine, DOPAC = 3,4-dihydroxyphenylacetic acid, 5-HIAA = 5-hydroxyindoleacetic acid. | | | | |

| **Supplementary Table 8. Significant differences in serum monoaminergic levels of DLB patients taking vs not taking antidementia medication** | | | | |
| --- | --- | --- | --- | --- |
| **Parameter** |  | **Antidementia medication**  **(n = 14)** | **No antidementia medication**  **(n = 30)** | **Mann-Whitney U statistics** |
| **DOPAC/DA** | Serum | 4.610  (2.716-12.308)  (n = 9) | 15.775  (6.090-76.634)  (n = 19) | *U* = 35.0  *P* = 0.013 |
| Data are presented as median with interquartile ranges between brackets. Mann-Whitney U tests were performed to discern differences in monoaminergic levels of DLB patients taking vs not taking antidementia medication. Statistical outcome is listed in the rightmost column. Abbreviations: DA = dopamine, DOPAC = 3,4-dihydroxyphenylacetic acid, DLB = dementia with Lewy bodies. | | | | |

| **Supplementary Table 9. Significant differences in serum and plasma monoaminergic levels of DLB patients taking vs not taking antiparkinsonian medication** | | | | |
| --- | --- | --- | --- | --- |
| **Parameter** |  | **Antiparkinsonian medication**  **(n = 13)** | **No antiparkinsonian medication**  **(n = 31)** | **Mann-Whitney U statistics** |
| **MHPG**  **(ng/mL)** | Plasma | 28.273  (17.010-38.705)  (n = 10) | 12.118  (3.178-25.472)  (n = 28) | *U* = 204.0  *P* = 0.034 |
| **DA**  **(ng/mL)** | Serum | 0.301  (0.103-0.568)  (n = 10) | 0.854  (0.521-1.201)  (n = 22) | *U* = 48.0  *P* = 0.012 |
| Data are presented as median with interquartile ranges between brackets. Mann-Whitney U tests were performed to discern differences in monoaminergic levels of DLB patients taking vs not taking antiparkinsonian medication. Statistical outcome is listed in the rightmost column. Abbreviations: DLB = dementia with Lewy bodies, DA = dopamine, MHPG = 3-methoxy-4-hydroxyphenylglycol. | | | | |

| **Supplementary Table 10. Significant correlations of the H/M ratio with CSF, serum and plasma monoaminergic levels of DLB patients free from antidepressants** | | | |
| --- | --- | --- | --- |
| **Parameter** |  | **DLB**  **(n = 18)** | **Correlation to**  **H/M ratio** |
| **H/M ratio** |  | 1.290  (1.145-1.685)  (n = 18) | / |
| **HVA**  **(ng/mL)** | CSF | 28.560  (17.250-44.649)  (n = 18) | ρ = 0.474  *P* = 0.047 |
| **5-HIAA/5-HT** | Plasma | 1.557  (0.467-4.890)  (n = 15) | ρ = -0.565  *P* = 0.028 |
| **HVA/5-HIAA** | Plasma | 0.978  (0.717-1.628)  (n = 18) | ρ = 0.621  *P* = 0.006 |
|  | Serum | 0.561  (0.295-1.138)  (n = 13) | ρ = 0.787  *P* = 0.001 |
| Data are presented as median with interquartile ranges between brackets. Spearman correlations tests were performed to correlate monoaminergic levels to the H/M ratio in DLB patients free from antidepressants. Statistical outcome is listed in the rightmost column. Abbreviations: CSF = cerebrospinal fluid, DLB = dementia with Lewy bodies, H/M = heart-to-mediastinum, 5-HIAA = 5-hydroxyindoleacetic acid, HVA = homovanillic acid, 5-HT = 5-hydroxytryptamine (serotonin). | | | |
